# Global expression analysis of EBV-infected B cells early and late after infection reveals a dynamic interplay between growth and survival signals

**DOI:** 10.1186/1750-9378-7-S1-P34

**Published:** 2012-04-19

**Authors:** Alexander Price, Jason Tourigny, Eleonora Forte, Micah Luftig

**Affiliations:** 1 Department of Molecular Genetics and Microbiology, Duke University, Durham, NC, USA

## 

Epstein-Barr virus (EBV) is a member of the γ-herpesvirus family estimated to infect 90% of the world’s adult population. Despite the high prevalence of infection, EBV-associated malignancies are largely kept in check by a strong cytotoxic T cell immune response. However, EBV causes lymphoproliferative disease in immune-deficient individuals following transplant and CNS and other lymphomas in HIV-infected individuals. EBV also plays a role in the pathogenesis of endemic African Burkitt’s lymphoma, Hodgkin’s disease, and nasopharyngeal carcinoma. *In vitro*, EBV infection of primary human B cells results in proliferation and outgrowth of indefinitely proliferating lymphoblastoid cell lines, or LCLs, which represent a viable model for the pathogenesis of EBV-associated malignancies.

Ongoing studies in our group have shown that the earliest EBV-infected proliferating B cells differ greatly from LCLs phenotypically. Using CFSE staining and flow cytometry-based sorting, we have isolated these early proliferating B cells and analyzed genome-wide exon level mRNA expression relative to uninfected resting B cells and LCLs. Gene ontology analysis of these expression data identified enrichment of genes associated with proliferation and the DNA damage response in early proliferation. Furthermore, c-Myc mRNA and activity, as inferred from its genome-wide expression signature, were also highly induced early.

Most interestingly, however, analysis of changes from early proliferating to final LCL outgrowth revealed striking attenuation of proliferative gene sets and c-Myc, along with delayed induction kinetics of NFκB activation. Specifically, genes with NFκB motifs in their promoters were highly expressed from early proliferating B cells to LCL and many canonical NFκB targets and pathway components were induced at late times after infection. These results suggest a novel, dynamic EBV-driven growth pattern and expression program that relies on mutually exclusive signals from c-Myc and NFκB. Furthermore, our data suggest that the earliest stages of EBV-driven B cell immortalization may provide unique insight into the pathogenesis of EBV-associated malignancies.

